# What causes severe malaria and its complications in children? Lessons learned over the past 15 years

**DOI:** 10.1186/s12916-019-1291-z

**Published:** 2019-03-07

**Authors:** Andrea L. Conroy, Dibyadyuti Datta, Chandy C. John

**Affiliations:** Ryan White Center for Pediatric Infectious Diseases and Global Health, Indiana University School of Medicine, 1044 W Walnut St R4 402D, Indianapolis, IN USA

**Keywords:** Severe malaria, Cerebral malaria, Severe malarial anemia, Pathogenesis, *Plasmodium falciparum*, Acute kidney injury, Cognitive, Neurodevelopmental, Impairment

## Abstract

Over the past 15 years, malaria mortality has reduced by approximately 50%. However, malaria still causes more than 400,000 deaths annually, most of which occur in African children under 5 years of age. Significant advances in understanding the pathogenesis of the disease provide a basis for future work to prevent severe malaria and its complications. Herein, we provide an overview of advances in our understanding of severe malaria in African children over the past 15 years, highlighting key complications and identifying priorities to further reduce malaria-associated mortality.

## Background

**Fig. 1:**
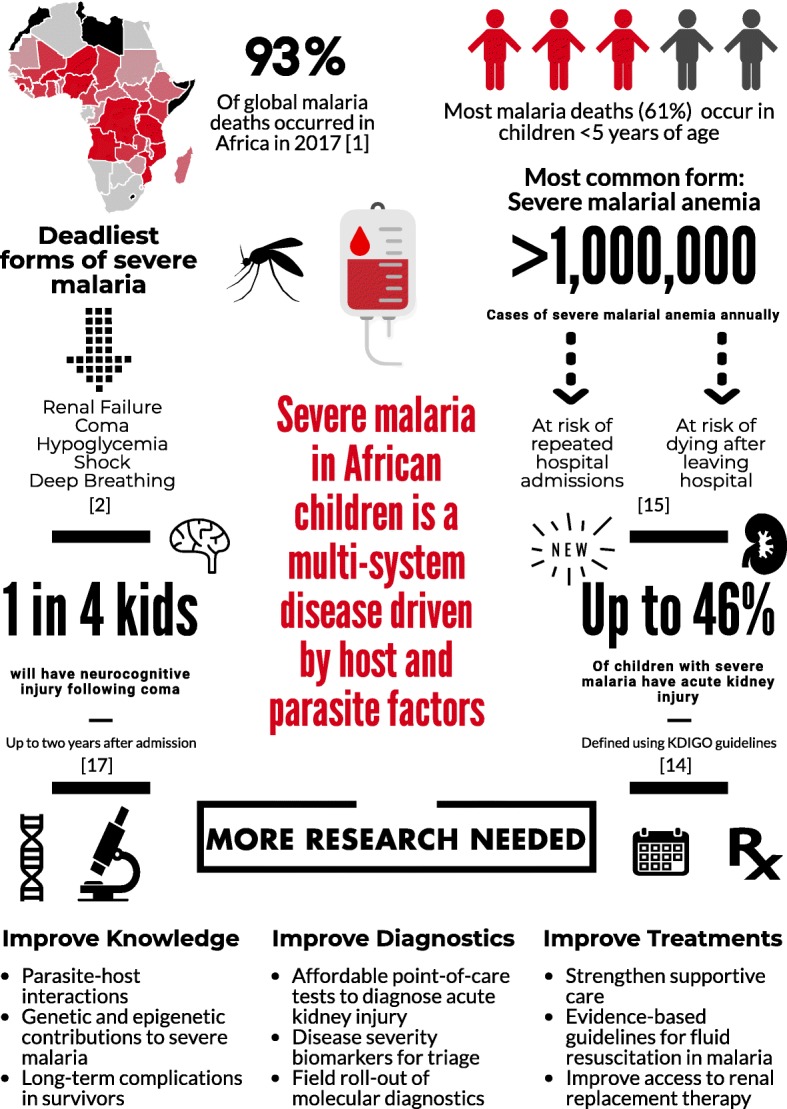
Severe malaria and its complications

*Plasmodium falciparum* accounts for the majority of malaria deaths, and is the predominant malaria species in Africa (Fig. 1) [1]. Severe malaria (SM) is defined by the detection of *P. falciparum* by microscopy or a rapid diagnostic test and at least one criterion for severe disease (impaired consciousness, respiratory distress, multiple convulsions, prostration, shock, pulmonary edema, abnormal bleeding, jaundice, severe anemia, hypoglycemia, acidosis, hyperlactatemia, renal impairment, or hyperparasitemia) [2]. Further, mortality can exceed 50% when multiple prognostic factors are present [3].

SM is a multi-system disease characterized by a systemic inflammatory response. A central feature in SM is the sequestration of parasitized red blood cells (pRBCs) in vascular beds, leading to impaired tissue perfusion and lactic acidosis. Parasite ligand *P. falciparum* erythrocyte membrane protein-1 (PfEMP1) is expressed on the pRBC membrane, where it binds to endothelial receptors (e.g., CD36, soluble ICAM-1). Recently, the discovery of endothelial protein C receptor (EPCR) as a novel and conserved host receptor for PfEMP1 binding transformed our understanding of SM pathogenesis (reviewed by Bernabeu and Smith [4]), providing a link between endothelial activation, inflammation, coagulation, and genetic susceptibility to SM driven by host and parasite genetics.

Endothelial activation is common in SM and is associated with upregulated cellular adhesion molecules on endothelium and their concomitant increase in plasma through ectodomain cleavage [5]. The angiopoietin-Tie2 signaling pathway has an important association with disease severity and mortality in pediatric SM [6], and has been implicated in blood–brain barrier breakdown and death in experimental cerebral malaria (CM) [7].

## Diverse forms of SM

SM is a multi-system disease driven by both host and parasite factors. CM is the deadliest form of SM. Children with CM often have malaria retinopathy, presenting with hemorrhages, retinal whitening, and vessel color changes, all of which can be visualized by trained observers using ophthalmoscopy. Interestingly, these changes mirror findings in the brain at autopsy [8]. Further, the development of radiologic capacity in low-resource settings has led to advances in our understanding of CM, with studies in Malawi showing that cerebral edema predicts mortality in CM [9] and is associated with EPCR-binding parasites [10].

Respiratory distress is a common form of SM best characterized by deep acidotic breathing. Like other forms of SM, respiratory distress is multifactorial. Impaired tissue perfusion secondary to pRBC sequestration and reduced oxygen-carrying capacity in severe anemia contribute to acidosis [11]. It is estimated that lactic acid contributes to half the acid load in SM, with several other organic acids being elevated in SM [12]. The kidney is important in acid metabolism and excretion, and may be involved in acidosis in SM. Additionally, respiratory distress and acute kidney injury (AKI) are linked by oxidative stress from the destruction of pRBCs and the release of free heme [13].

Fifteen years ago, renal failure was considered a rare complication in children with SM, yet it is now recognized as one of the strongest predictors of mortality in SM [2]. The recognition that small changes in kidney function independently predict mortality in critical illness led to the development of new guidelines to define AKI (Kidney Disease: Improving Global Outcomes, KDIGO) [14]. In a prospective cohort of Ugandan children with SM, AKI was common, occurring in 46% of young children with SM [15]. Although data suggest AKI is related to reduced kidney perfusion, additional studies are needed to evaluate the spectrum of AKI over hospitalization to define the etiology and pathophysiology of AKI in pediatric SM.

Severe malarial anemia (SMA) is the most common form of SM. The etiology of SMA is complex, involving increased destruction and removal of infected and uninfected RBCs, and reduced RBC production due to bone marrow dyserythropoiesis (recently reviewed by White [16]). SMA can occur in the absence of other SM complications in children with repeated or inadequately treated infections, and mortality is low with appropriate transfusion [11]. However, SMA is not benign – it contributes to significant long-term morbidity, including impaired neurocognitive functioning [17], repeated hospitalizations, and post-discharge mortality [16].

## Long-term complications associated with SM

One in four children prospectively enrolled in studies with CM develop neurocognitive impairments that persist at least 2 years following exposure [18]. Retrospective studies suggest impairments may last 8 years or longer, and may include behavioral problems, mental health issues, and the development of epilepsy (reviewed by Idro et al. [19]). Children with SMA have long-term complications related to cognition [17]; therefore, given the huge burden of SMA, it may be a significant contributor to neurocognitive impairment in African children. Nevertheless, the mechanisms leading to brain injury and subsequent neurocognitive complications due to SM are not well understood. In particular, questions remain about how an intravascular parasite can lead to such a breadth of complications, and why these complications are only observed in a fraction of the population with SM [19].

Elevated cerebrospinal fluid (CSF) levels of TNF-α are associated with prolonged coma duration, neurologic deficits, and long-term cognitive deficits in children over 5 years [20]. A strong correlation of the CSF-to-plasma TNF-α ratio and CSF-to-plasma albumin index (an indicator of blood–brain barrier impairment) suggest a degree of permeability across the blood–brain barrier [20]. Metabolites of the kynurenine pathway are markedly elevated in the CSF of children with CM and have been associated with prolonged coma and impaired attention in children over 5 years [19, 21]. Axonal injury marker tau is elevated in CSF of children with CM and is associated with acute neurologic deficits (reviewed by Idro et al. [19]). Our studies in Ugandan children with CM suggest elevated CSF-tau is associated with prolonged coma duration and long-term cognitive impairment, particularly in children over 5 years, and may be mediated in part by blood–brain barrier impairment. Additional studies are needed to delineate the mechanisms leading to neurocognitive complications in SM, particularly in children without overt clinical signs suggestive of brain injury.

## Conclusions

Figure 1 outlines the burden of SM and the ways in which this burden could be lessened in the future. Prevention of SM and its complications will require better implementation of known preventive measures, including primary measures to prevent infection (e.g., insecticide-treated bed-nets) and secondary measures to prevent severe disease such as rapid access to care, use of appropriate malaria diagnostics, and effective treatment of uncomplicated malaria. Increased knowledge of clinical prognostic signs and implementation of point-of-care tools to identify children at risk of clinical deterioration or death could facilitate directed use of intrarectal artesunate in primary health centers prior to referral and transport to tertiary centers. Better knowledge on pRBC and endothelial cell interactions – in particular ICAM-1 and EPCR-binding parasites – may lead to novel vaccine targets. Further, research on the etiology and pathogenesis of AKI in SM, an important complication that remains poorly understood, is critical. Point-of-care tools to facilitate prompt recognition of AKI, development of resource-appropriate kidney care guidelines, and early referral to higher levels of care could reduce the long-term impact of AKI on children’s health. Finally, a better understanding of the pathogenesis of neurodevelopmental complications, as well as the long-term health costs of these complications, may lead to interventions to reduce neurodevelopmental disability in survivors. In the long run, investment in both primary malaria prevention and better management of SM will lead to substantial health benefits for children in sub-Saharan Africa.
